# Intercorporeal Formations in Pediatric Dental Encounters With Patients Showing Distress: The Intertwine of Controlling and Comforting Touch

**DOI:** 10.1177/10497323231211451

**Published:** 2023-11-20

**Authors:** Julia Katila, Enhua Guo, Niaz Aziz, Katie E. Bradford, Satu Lahti

**Affiliations:** 1 Faculty of Social Sciences, 7840 Tampere University, Tampere, Finland; 2 School of Foreign Languages, 12591 Ocean University of China, Qingdao, China; 3 Department of English, 502623 Soran University, Erbil, Iraq; 4 Department of Communication Studies, 12330 The University of Texas at Austin, Austin, TX, USA; 5 Department of Community Dentistry, 8058 University of Turku, Turku, Finland

**Keywords:** touch, pediatric dentistry, dental fear, intercorporeality, video analysis, interaffectivity, embodiment, bodily experiences

## Abstract

Dental fear and anxiety are highly prevalent among children and have been shown to lead to irregular use of dental services. Previous research has suggested that while touch can alleviate the patient’s stress and help in accomplishing dental procedures, it can also be a source of stress or used to restrain the patient. In this study, we explore the emergence and intertwine of controlling and comforting touch in pediatric dental clinic settings in which patients show signs of resistance, distress, or fear. We use microanalysis of video-recorded interactions to unveil how the adults in the room—any combination of the dentist, dental assistant, hygienist, and caregiver(s)—deploy various types of touch on the child patient to perform the dental procedure while simultaneously comforting the child. Our data set covers video-recordings of naturally occurring dental clinic visits of 3- to 12-year-old child patients from four cultural contexts: Finland, China, Iraq, and the United States. Drawing on Merleau-Ponty’s writings on intercorporeality and the interaffectivity of bodies, the study proposes that touch in pediatric dentistry unfolds as complex intercorporeal formations where the interaffectivity emerges not only through touch but also via vocal resonance. In contrast to clear boundaries between comforting and controlling touch, our analysis indicates that the line between comforting and controlling touch can be blurred. We suggest that touching a pediatric patient showing resistance toward a dental procedure requires careful affective attention to the patient’s subtle and moment-by-moment bodily expressions and reactions to the touch.

## Introduction

Touch is a body-to-body act, directly influencing human beings’ embodied, sensorial experience. For instance, in the wake of pain, ache, or disease, therapeutic touch has the ability to alleviate human suffering (Anderson & Taylor, 2011; Beach, 2022; Oliveira et al., 2017; Paterson, 2005). Previous studies have established the positive consequences of interpersonal touch: it is beneficial for people’s health and wellbeing (e.g., Field, 2014; Triscoli et al., 2017) as well as a primordial medium for interpersonal communication and connection (Goodwin & Cekaite, 2018; Katila, 2018; Montagu, 1986). However, the power of touch is not limited to positive consequences; touch can also cause hurt and pain or increase a person’s distress, as in the case of domestic violence or abuse (e.g., Malinosky-Rummell & Hansen, 1993; Stover, 2005). In health care contexts such as that of pediatric dentistry, the professionals touch the patients in various ways, both skin-to-skin and tool-mediated, to conduct the institutional tasks of examining, caring for, and treating patients, and these ways of touching may cause an array of bodily sensations in the patients from the sensation of ease and comfort to distress and pain.

Previous research has established that touch can help in the accomplishment of dental procedures. For example, Greenbaum et al. (1993) found that pediatric patients who were patted gently on their upper arm or shoulder during an examination displayed less fidgeting behavior than their no-touch counterparts. However, besides calming, in some situations, touch is used to constrain the patient (cf. Davidhizar & Newman, 1997). Kent (1984) showed that holding and restraining children increased their fearful responses, while patting seemed to reduce such responses. In the context of pediatric dentistry, Guo et al. (2020) suggested that controlling touch conducted on a patient could blur bodily boundaries of participants since the bodily agency—the capacity to initiate and control one’s body—of the child was distributed among the touching participants.

This study examines touch in dental clinic settings in which pediatric patients show signs of resistance, distress, and fear as they interact with various adults—dentists, hygienists, dental assistants, and caregivers—and the adults use touch to mitigate the child patients’ expressed concern or anxiety while also working to complete the dental treatment. Our data set covers video-recordings of dental clinic visits of 3- to 12-year-old child patients from four cultural contexts: Finland, China, Iraq, and the United States. We use microanalysis of the video-recorded interactions (e.g., Heath et al., 2010; Streeck et al., 2011) to uncover the various types of touch the adults in the room use with the patients in order to perform the dental procedures while simultaneously addressing children’s emotional needs. Video analysis of interaction is a systematic approach that unveils the details of naturally occurring, unscripted interactions. As such, it is especially suitable for capturing the details of interaction that might be hard to memorize or verbalize afterward, such as specific ways of touching and corresponding reactions to touch.

Our study identifies, distinguishes, and describes the nuances and complexities of touch in pediatric dental appointments. In previous literature, affectionate, gentle, tactile actions have been characterized by numerous terms, depending on the field and context: caring (Raia et al., 2020), compassionate (Burdelski, 2020), affectionate (Bergnehr & Cekaite, 2018; Mononen, 2019), reassuring (Greenbaum et al., 1993), and therapeutic (Monroe, 2009; Randolph, 1984). In their research on adults’ responses to crying children, Cekaite and Kvist Holm (2017) use the term *comforting touch* to describe gentle, body-to-body contact aimed at soothing or calming. In this study, we use the term *comforting touch* more generally to refer to touch that encompasses all of above meanings. And we use the term *controlling touch* to refer to restraining and sustaining tactile actions by individuals who manage and monitor another person’s body (Cekaite, 2015, 2016). While our data includes clear cases of both controlling touch and comforting touch, touch often occurs along a continuum, escaping the clear labeling and coding expected from a scientific study (cf. MacLure, 2013). Finally, we use the term *procedural touch* to refer to the task-oriented touch of the dental team (Goodykoontz, 1980; cf. Cocksedge et al., 2013; Routasalo, 1999).

The mouth is one of the most densely innervated parts of the body and thus also is quite sensitive to touch (Haggard & de Boer, 2014). When children experience fear, pain, anxiety, or discomfort, their bodily reactions are not necessarily under their control, especially given the sensitive body part being touched during the dental treatment. This not only leads to an uncomfortable situation for the child but also may preclude or endanger safe and appropriate performance of dental procedures. Dental fear and anxiety are highly prevalent among children and adolescents (Cianetti et al., 2017; Grisolia et al., 2021), are associated with poorer emotional and social dimensions of oral health-related quality of life (Pohjola et al., 2009), and have been shown to lead to non-habitual use of dental services (Liinavuori et al., 2019). As such, childhood interactions at the dental clinic may have a long-lasting influence on individuals’ willingness to participate in dental care. Therefore, uncovering the interplay of comforting and controlling touch in this context helps us to better understand the interaction involving dental treatment of children who are distressed or fearful, and how these situations could be dealt with by showing effective care and compassion toward patients.

Theoretically, we approach the manifold aspects of touch through the lens of the *intercorporeality* of bodies—a theory about the primacy of human embodiment, developed by phenomenologist Merleau-Ponty (1962, 1964, 1968). Intercorporeality refers to the ability of humans to experience meaning together through their affective bodies. Touch unites bodies in an intrinsic intercorporeality (Merleau-Ponty, 1962): one cannot touch without being touched; thus, touch enables direct body-to-body communication at a pre-reflective and pre-discursive level. Further, given that touch affects the human bodily experience directly—such as through soothing, controlling, or caring—from the intercorporeal perspective, the affectivity^1^ of touch becomes *interaffectivity* (Fuchs, 2017). This means that as much as one cannot touch without simultaneously being touched, when touching, one also cannot affect without also being affected. The notion of interaffectivity explains affect as “primarily residing not within a single individual, but as phenomena of a shared intercorporeal space in which the interactants are involved” (Fuchs, 2017, p. 4). In her phenomenological inquiry on nurse’s touch in a neonatal intensive care unit, Lemermeyer (2021) concludes, “The nurse is not only the one who touches the baby but also the one being touched by the baby, in the inherently ethical experience of touch—making relational contact with another” (p. 1578). In the context of the current study, interaffectivity of touch becomes especially salient in the patient’s bodily reactions to the dental procedures. As we will show, an intercorporeal perspective to the affectivity of touch challenges the clear separation between the self and the other that has often been the traditional starting point for an analysis of touch in interaction (Montagu, 1986; Josipovici, 1996; also cf. Tahhan, 2013).

## Data and Method

The study data includes video-recordings of dental clinic visits of 3- to 12-year-old child patients with signs of dental fear in China, Finland, Iraq, and the United States. Extracted from a wider data set consisting of 143 dental appointments, our collection covers 39 (9 in Finland, 11 in China, 14 in Iraq, and 5 in the United States) appointments with patients who show signs of distress, fear, pain, or expression of embodied resistance toward the anticipated or ongoing procedure. To our knowledge, similar studies uncovering the emergence of touch in naturally occurring pediatric encounters have not been done before.

The dental fear of patients in this study was not measured. Rather, we selected the examples used in this study based on our empirical observations of the child–hygienist or child–dentist interactions, choosing cases where patients show resistance, discomfort, or signs of fear, such as through crying, breathing heavily, negative facial expressions, or a rigid bodily stance. Often, the patients also explicitly verbalized their discomfort or fear before or during the procedure.

The data collection in each country carefully followed the ethical regulations of each country. Before data collection, relevant ethical committee permissions were obtained, and written informed consent to participate in the study was obtained from the patients and their caregivers. To protect the identities of the participants, pseudonyms are used, and figures from the data are drawn using Photoshop. The verbal transcription conventions are presented in Appendix 1. The conventions are modified for our purpose from the work of conversation analysts Gail Jefferson (Sacks et al., 1974, pp. 731–733) and Kang-Kwong Luke (2019).

We use microanalysis of the video-recorded interactions (Heath et al., 2010; Streeck et al., 2011), which involves detailed analysis of the moment-by-moment emergence of touch and how it is interpreted by the participants themselves. Importantly, video analysis focuses not only on touch in isolation but also on all other multimodal behavior occurring during the moments of touch, including gaze, gestures, spoken words, and tone of voice. These modalities and senses mutually elaborate each other (Goodwin, 2000), constituting a multimodal gestalt (Mondada, 2018). Moreover, we analyze how multiple various types of touches by different participants sometimes occur simultaneously. For example, the dentist’s or hygienist’s procedural touch is often accompanied by comforting and controlling tactile actions by the assistant or the caregiver. As such, we consider touch in interaction through the lenses of intercorporeal formations, including multiple participants and multimodal (i.e., diverse communication modes) and multisensorial (i.e., related to different senses) resources.

Rather than focusing on potential cultural differences in touching practices among Finnish, Chinese, Iraqi, and U.S. dental health care contexts, we analyze salient common features we find in all these settings: the emergence of comforting and controlling touch, and the fact that they are often intertwined and co-occurring with the discomfort-causing procedural touch of the dental care providers. The four episodes selected for analysis and discussion (Extracts 1–4) are representative of the observed variations in the comforting–controlling tactile practices found in our collection.

When applicable, we complement the video analysis with our field observations and conversations or interviews with the dental professionals. Because authors 1–4 each collected data from their home countries, we are able to contribute knowledge of the specific local cultural context and language. Moreover, in our analysis, we utilize the professional vision of a dentist, as one of the authors is a former practitioner with 25 years of experience in treating patients with dental fear and is currently working as an educator of dentists and dental students.

## Analysis

In the following analyses, we demonstrate the role of comforting and controlling touch in accomplishing the institutional tasks of pediatric dental treatment when patients show signs of discomfort, resistance, or fear. We will illustrate that (1) both comforting and controlling touch are intercorporeal acts that directly affect the patient’s body; (2) comforting and controlling touch occur as intercorporeal formations, often participated in by more than two bodies; (3) comforting and controlling aspects of touch often are intimately intertwined in the pediatric dental context; and (4) the meaning of touch often occurs in conjunction with other embodied modalities.

### Comforting Touch as an Interaffective Resource in Distributing Corporeal Calmness and Reassurance

Previous studies have suggested that comforting touch involving embraces, stroking, and patting is often used to comfort a body under stress (e.g., Cekaite & Kvist Holm, 2017; de Leon, 2021; Raia et al., 2020). In Extract 1, recorded in a Finnish context, we show how comforting touch can be harnessed to alleviate a child’s fear or pain brought about by procedural touch. We suggest that comforting touch in this context can be viewed through the lens of the interaffectivity of bodies: it is the bodies’ way to distribute calmness from one body to another and neutralize the influences of potentially fearful or hurtful touch. In addition, comforting touch can be augmented by verbal actions with a soothing tone of voice. As the dentist, dental assistant, and mother soothe the patient with touch applied to different parts of his body, the bodies together form an intercorporeal formation connected through the multiparty touch.

**Figure 1.**
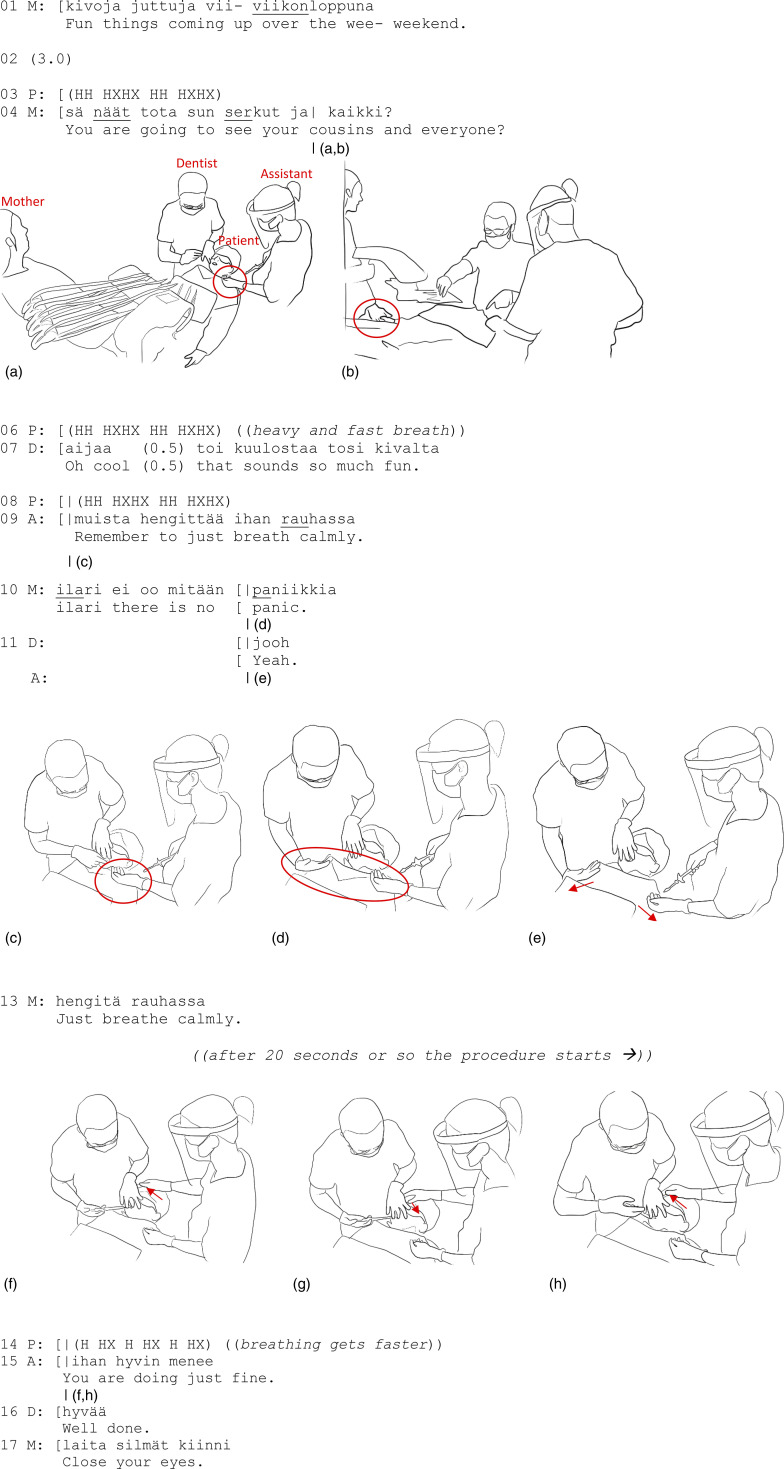
(a, b) A touches P with the left hand. (c) A touches P once again with the back of the hand. (d) D and A start a synchronized touch. (e) D caresses P with the back of her left hand. (f–h) A holds P’s head with the right hand and caresses him with the thumb.

As the extract starts, the dentist is about to give the patient, an 8-year-old boy, a local anesthesia injection (Figure 1(a)). The mother holds the patient’s hand and continuously gently rubs the patient’s hand with her thumb (Figure 1(b)), providing an ongoing tactile medium for being with her child (cf. Goodwin, 2017, p. 76), a tactile means of doing togetherness.

While the dentist prepares the syringe, the mother talks to her son about the forthcoming fun weekend with his cousins, presumably trying to distract him from the present uncomfortable moment (lines 01 and 04). The dentist, supporting the mother’s action, provides an appreciative response to this (oh cool (0.5) that sounds so much fun, line 07). The patient does not attend to the mother’s and dentist’s actions but begins fast-paced breathing to the point it is clearly audible, and his body visibly shivers (lines 03, 06, and 08). The assistant takes note of this and touches the patient’s chest lightly with her fingers (Figure 1(c)), at the same time verbally encouraging him to breathe calmly (line 09). Immediately following this, the dentist and the mother also attend to the patient’s emerging distress and provide reassuring utterances (lines 10–11 and 13). Almost in synchrony, the dentist and assistant touch the patient, with the assistant on the left side of the patient’s chest and the dentist on the equivalent right side of the patient’s chest while still holding the patient’s mouth open with her left hand (Figure 1(d) and (e)). By using the dorsal aspect of the hand rather than the palm of the hand, these instances of touch appear to be comforting—affectionate and soothing—rather than controlling. Produced together, these tactile actions distribute corporeal reassurance to the patient’s body, attending to the patient’s stress.

After 20 seconds or so, the actual procedure starts. As the dentist uses her right hand to give the injection and left hand to hold the patient’s mouth open, the assistant continues holding her left hand on the patient’s chest (Figure 1(f)). Moreover, the assistant moves her right palm to the patient’s head and gently caresses his forehead with her thumb (Figure 1(f)–(h)). At the same time, the three adults continue their reassuring verbalizations (lines 15–17), and the mother continues holding her son’s hand. For a moment, the adults focus only on soothing the patient’s feeling body, via a multimodal gestalt, combining both verbal reassurances and comforting touch.

In Extract 1, we demonstrate that comforting touch can help a patient remain still during an otherwise uncomfortable situation. The needle penetrates the skin, and the body must for a moment simply surrender to the discomfort or pain caused by the injection. Despite his fast-paced breathing, the patient manages to cooperate and keep his body still. Multiple, simultaneously-occurring types of touch and verbalizations of different adults together are there to affect the patient’s body with a comforting resonance that balances the uncomfortable sensation.

While the feelings and bodily sensations during this procedure are only felt by the patient who experiences them, other participants provide their tactile co-presence and forms of comforting touch, easing and supporting the dental patient. During the moment of gentle soothing, the one performing the soothing momentarily lends her or his body to the one being soothed: through touch, they intercorporeally take part in the other’s bodily sensations and feelings, even if they are not directly feeling these sensations, as they empathize with the other. As suggested by Wyschogrod (1981), empathy is a feeling-act that brings the self nearer to the other. As such, empathy is not a mental act but rather an affective bond or emotional connection that is particularly embodied in touch. As Wyschogrod (1981) states with regard to various human senses, it is “only touch [that] requires contact, the proximity of feeling to what is felt” (p. 25). That is, touch-based experiences always require a haptic, immediate, physical contact between the body and the object (or the other body) being felt. Thus, comforting touch is a compassionate engagement resulting from empathy, an engagement that enmeshes the bodily boundaries and, in doing so, enables soothing another body in stress.

### Extended Touch as a Means of Achieving Intercorporeal Companionship

**Figure 2.**
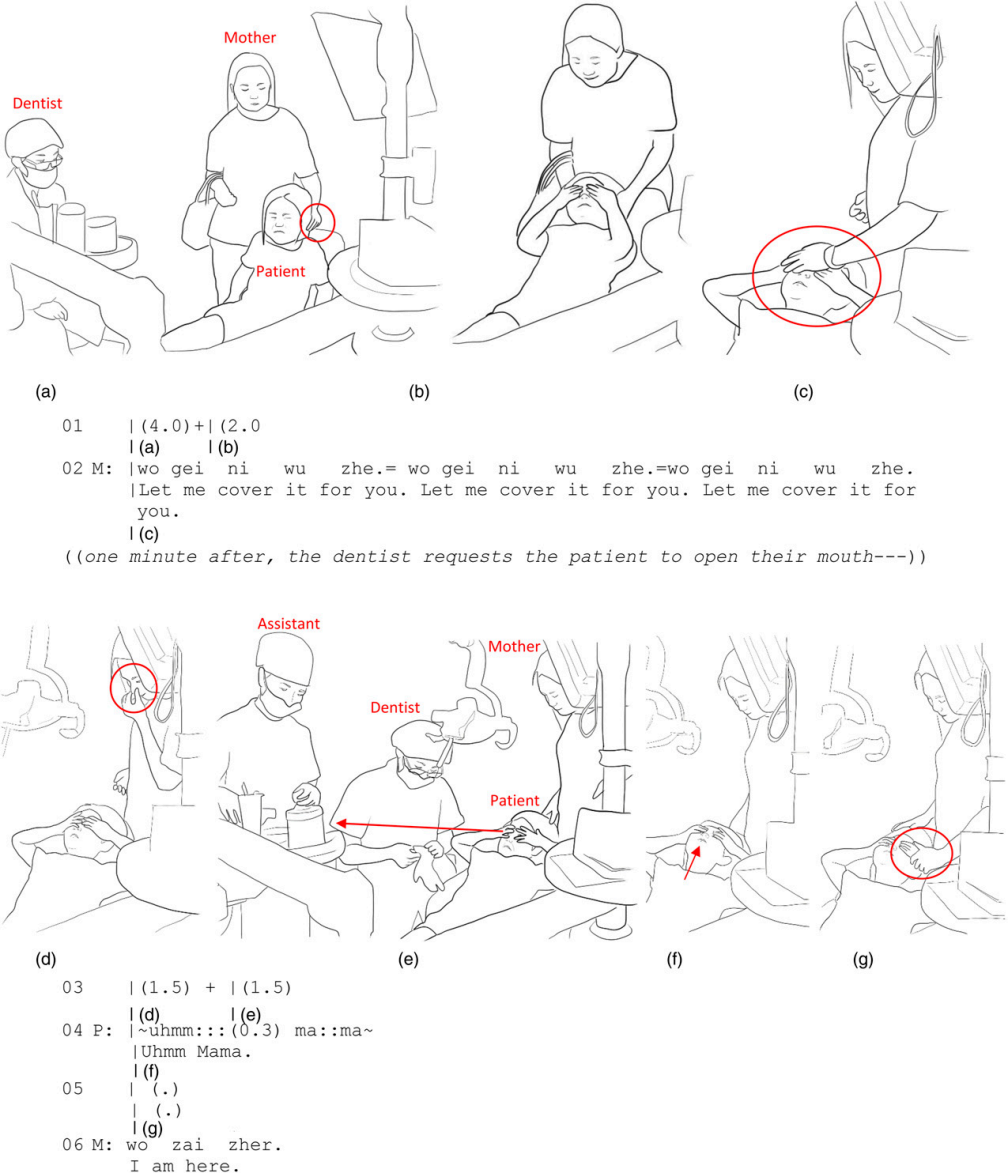
(a) P moves to the chair. (b) P closes her eyes and covers them with both hands. (c) M places her left hand on top of her daughter’s hand. (d) M withdraws her left hand. (e) P peeks through her fingers. (f) P covers her eyes again and raises her chin toward her mother. (g) M’s left hand swiftly grasps her daughter’s wrist.

Extract 2, recorded in China, demonstrates how extended comforting touch serves as a corporeal means of mediating co-presence amidst fear and therefore can act as a way of providing intercorporeal companionship in and through a difficult situation. In Extract 2, a 6-year-old girl has come to the clinic with her mother for a tooth extraction. Her mother uses gentle touch applied to the back of her daughter’s head to guide her to climb onto the dental chair (Figure 2(a)).

The patient gets onto the dental chair reluctantly with a fearful grimace on her face (Figure 2(a)). She then proceeds to cover her eyes through self-touch, employing her own hands to shield herself and block her visual access to the dental procedure, possibly as a way of distancing herself from the fearful situation. The mother immediately attends to this by touching the sides of her daughter’s head (Figure 2(b)). She then proceeds to place her hand on top of the child’s hands (Figure 2(c)) while soothingly saying, “Let me cover (your eyes) for you” repeatedly (line 02).

This touch, accompanied by the verbal reassurance, acts to corporeally support the child’s self-touch. While the girl already is doing a good job of covering her own eyes, the mother’s touch enhances this self-touch, providing the child with reassurance of the mother’s co-presence (Figure 2(c)). It is a means for the body to communicate “I am here for you” by providing an intercorporeal companionship through touch. To use Merleau-Ponty’s (1964, p. 168) expression, the hands of the mother and child are *compresent*, inhabiting a single intercorporeality. The hands of both the mother and the child are not only physically in contact, but they are coexisting in a way that reflects a unified bodily experience, as if they are sharing the same embodied reality. The motionless touch functions intercorporeally, transmitting a sense of calmness from one body to the other.

Moments later, the mother withdraws her hand to rub her nose (Figure 2(d)). As the mother does not immediately return her hand to touch her daughter, the child is left to cover her eyes on her own. Peeking through her fingers, the girl glimpses the dental team getting ready for the procedure: the assistant is preparing equipment, and the dentist is putting on gloves (Figure 2(e)). The sight of unfamiliar and intimidating dental equipment in the hands of the assistant may appear frightening to a child. In response to what she has seen, the young patient quickly covers her eyes once more and makes an audible expression of discomfort, calling out for her mother (line 04, Figure 2(f)). The mother immediately grasps her daughter’s hand (Figure 2(g)) and softly assures her, saying, “I am here” (line 06). Therefore, in this instance, touch provides tangible, intercorporeal evidence of the mother’s compresence given that the child, with her eyes covered, is momentarily unable to see her mother. This touch provides support through compresence combined with verbal action (Figure 2(g), line 06).

The analysis of Extract 2 shows that prolonged touch affords a medium for being with another person that cannot be entirely replaced by words alone. As Merleau-Ponty (1962) puts it, “through gestures my intention is inhabited by your body, while your intention inhabits mine” (p. 185). In Extract 2, This phenomenon becomes especially salient, as the child’s seeking support is directly connected with her mother’s intention to provide it, and vice versa. Through their intercorporeal connection, the child can feel that she is not alone in the fear-inducing moment; rather, she is with her mother. Therefore, a tactile, intercorporeal companionship is achieved.

### A Primarily Controlling Touch That Affords Trust and Comfort

In Extract 3, recorded in an Iraqi context, we explore further the interplay of controlling and comforting touch, and especially how the controlling touch can potentially imply a comforting aspect. While in most cases it is the dental team member or the caregiver(s) who touches the child, the roles also can be reversed, and the child can touch the adult back. We illustrate this by analyzing how the act of touching or pressing another body tightly can provide the child with a resource for coping with uncomfortable sensations caused by a procedure. In the extract, a five-and-a-half-year-old girl comes to the clinic accompanied by her mother. She has a cavity in her tooth, and the dentist is using a drill to clean it.

**Figure 3.**
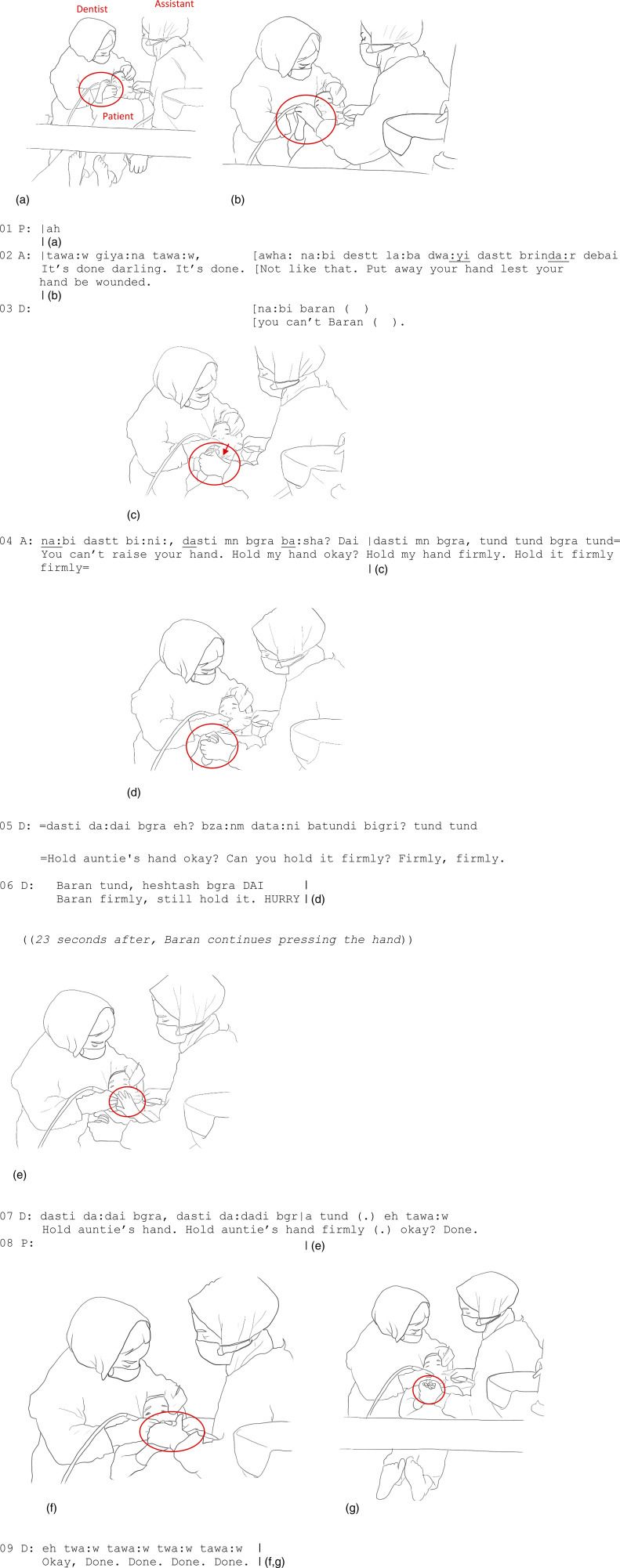
(a) P raises her right hand. (b) A grasps P’s hand. (c) A holds P’s hand. (d) A holds P’s hand. (e) P raises her left hand. (f, g) A encloses both P’s hands into hers.

The haptic negotiation between the child and the dental assistant begins with the assistant controlling the girl’s hand but slowly emerges into an affectionate huddle where the hands come together in a close bundle, both pressing each other tightly. In Figure 3(a), the girl, who has her eyes closed, produces a soft vocalization (line 01) and moves her right hand toward her mouth as a protective move. As an immediate response, the assistant, participating in the dental operation by using the suction in the girl’s mouth, grasps the girl’s hand (Figure 3(b)) while verbally foreshadowing the end of the treatment and directing the patient to put her hand away (line 02). Building on the dentist’s disallowing action (“you can’t Baran,” line 03), the assistant starts her next utterance once more, telling the girl that she should not raise her hand, but then the assistant continues her utterance with an alternative suggestion (“hold my hand firmly hold it firmly firmly,” line 04). At the same time, the assistant, taking the girl’s hand into hers, gently escorts it away from her mouth (Figure 3(c)). As their hands intertwine, the assistant encourages the girl to grab her hand even more firmly (line 06) and can witness the little hand pressing the assistant’s hand even tighter (Figure 3(d)).

Even if the assistant repositions the patient’s hand, the controlling haptic action entails affection. The assistant’s hand provides the child with a firm and safe basis to press as forcefully as she can and therefore a potential to channel the discomfort caused by the operation to the assistant’s hand. The assistant, holding the child’s hand tightly, also enables a form of corporeal security, affording the feeling of co-being in the difficult situation with another person. As such, we argue that controlling as it is, the assistant’s touch here provides the child with a feeling of “corporeal trust” (Cekaite & Kvist Holm, 2017)—a sense of reliance rooted in the assistant’s bodily support—that can ease the child’s discomfort. The patient responds to the assistant’s touch positively, manifested in her pressing the assistant’s hand continuously for another 23 seconds or so. The dentist then, once more, encourages the patient to “hold auntie’s hand firmly” (line 07). As the dentist continues with encouraging words about how the operation is almost done (line 09), the girl raises her left hand as well, reaching toward the assistant’s hand (Figure 3(e)). Attending to this corporeal request right away, the assistant also grasps the girl’s left hand (Figure 3(f)) and repositions it into the same secure bundle with the right hand (Figure 3(g)). The girl, looking calm while pressing the assistant’s hand, can manage through the operation.

In Extract 3, we witness a close co-emergence of controlling and comforting touch in soothing the body of a fearful child and an intertwining of the assistant’s hand and child’s hand as they both affect each other. The moment resembles what Merleau-Ponty (1968) has called a “chiasm,” or the “reversibility” of one person’s body and the body of another person, the coexistence of being with another person. In this example, the hands of both the assistant and the patient are simultaneously tangible to one another: they each feel their own hands touching themselves while also feeling the touching of the other person. As Merleau-Ponty (1968, p. 160) expresses, “...we situate ourselves in ourselves *and* in the things, in ourselves *and* in the other, at the point where, by a sort of *chiasm*, we become the others and we become world” (emphasis in original). Besides the reversal of touching and being touched, in Extract 3, the chiasm also implies a complex fission of controlling and comforting aspects of touch: it is neither just controlling nor just comforting but both together. This speaks to the unique and context-specific nature of forms of touch in our data that often escape clear labeling. In addition to gentle stroking or petting to provide comfort, sometimes a firm touch in the form of gentle control can provide the patient with a sense of protection.

### Controlling Touch Accompanied by Affective Words

In Extract 4, recorded in the U.S. context, we turn to a case that exemplifies controlling touch and illustrates the crucial role of verbal and vocal action in interpreting the meaning of touch. A 3-year-old girl has come to the dental clinic with her mother for her regular preventive-care visit, and the task at hand is to brush the girl’s teeth. A pediatric hygienist orchestrates the action using a combination of controlling and procedural touch, along with an affectionate tone of voice—that is, one that conveys care and affection—to verbally persuade the child to keep her mouth open for the procedure. The child displays discomfort through embodied resistance and crying but also agrees to participate in the brushing action by holding her mouth open and her body still.

**Figure 4.**
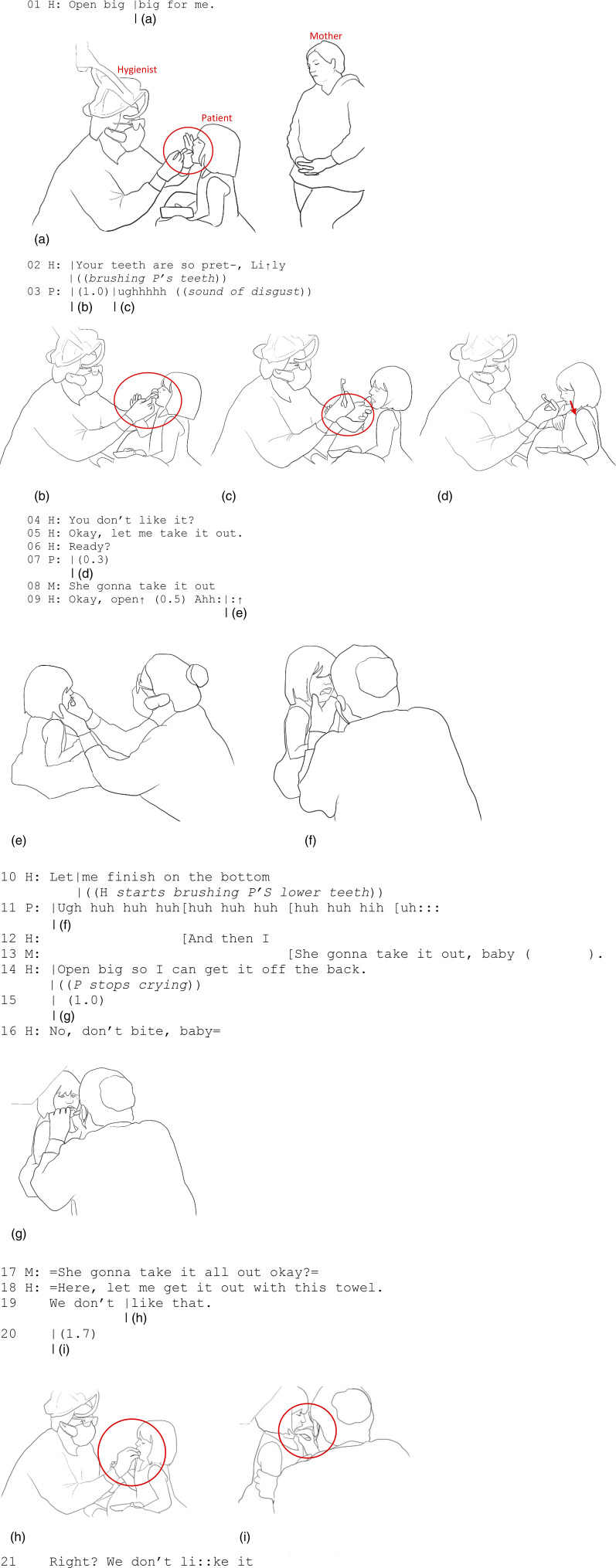
(a) H’s hand goes to P’s face, lifting up her upper lip. (b) P leans back, the right hand goes up to stop toothbrushing, and the right hand pushes H’s arm to the left. (c) H puts her left hand on P’s right arm. (d) P’s mouth is shut in resistance. (e) P opens her mouth. (f) P starts crying. (g) P closes her mouth on H’s finger. (h) H’s hand with cotton gauze goes into P’s mouth. (i) H starts wiping P’s mouth.

In Extract 4, we observe a subtle embodied negotiation between the hygienist and the young patient over the accomplishment of the brushing activity. This negotiation involves multiple evolving forms of touch as well as careful coordination between talk and touch. Using an encouraging tone of voice, the hygienist asks the patient to open her mouth (“Open big-big for me” in line 01) while gently lifting the patient’s jaw and using her fingers to help the patient open her mouth (Figure 4(a)). These tactile actions co-occurring with the verbal request guide the patient’s mouth to an appropriate position to accomplish the task at hand. Therefore, the use of touch affects the patient’s body by controlling her movement trajectory, yet most of the motor control remains with the patient, who voluntarily opens her mouth in a manner guided by the hygienist’s touch.

Starting the brushing movement, an instrument-mediated touch, the hygienist praises the appearance of the patient’s teeth in an affectionate tone (“Your teeth are so pret-, Li↑ly” in line 02). A moment later, the patient leans her body backward in resistance; at the same time, her right hand goes up and moves the hygienist’s hand away to defend herself from the brushing action (Figure 4(b)). She produces a vocalization of disgust (“ughhhhh” in line 03; see Goodwin et al., 2012) while her facial expression also expresses disgust. The hygienist verbally attends to the patient’s reaction of disgust, asking, “You don’t like it?” (line 04), and moves the back of her left forearm and hand—the hand that was just holding the patient’s mouth in position—on top of the patient’s right hand to gently control it and move it out of the way so she can continue the cleaning (Figure 4(c)). These instances of touch, first by the patient and then by the hygienist, form a sequence of gentle haptic negotiation over the position of the hands, in the sense of putting the patient’s unsolicited initiative on hold (Cekaite & Bergnehr, 2023). Thus, in the moment, both participants affect and are affected by each other through the corporeal force of the controlling touch: the patient’s first touch moves the position of the hygienist’s hand, while the hygienist then lightly controls the patient’s hand by guiding it away from hers.

Next, the patient responds by continuing her grimace and closing her mouth and turning her head downward (Figure 4(d)). The hygienist continues, “Okay, let me take it out” (line 05), with “it” referring to the thing the patient does not like, that is, the toothpaste or the dental plaque in the patient’s mouth that needs to be taken away by toothbrushing. Here, unlike Extracts 1, 2, and 3, where talk mainly appears in the form of directives, at line 05, the hygienist’s verbal announcement frames her incipient toothbrush-mediated touch within a larger operative framework (Cekaite & Bergnehr, 2023) of toothbrushing. Through these verbal actions, the hygienist conveys her understanding of the patient’s disgust and persuades the girl to allow her to continue brushing. The hygienist does so by positioning herself as someone who is “on the patient’s side,” the one who is going to ease the patient’s negative sensations of disgust by continuing, not stopping, the brushing. The hygienist then asks the patient, “Ready?” (line 06), but the patient responds with a shut mouth (Figure 4(d)). At this point, the girl’s mother, who stands to the side, leaning against a nearby wall and observing the interaction, supports the hygienist’s action, saying, “She gonna take it out” (line 08). The hygienist asks the girl to open her mouth (“Okay, open, Ahh” in line 09), to which the girl finally aligns (Figure 4(e)).

As the hygienist starts brushing the patient’s teeth again and encourages the patient to let her “finish on the bottom” (line 10), the patient starts crying (line 11, Figure 4(f)). Her mother again attends to her, saying in an empathetic tone, “She gonna take it out, baby” (line 13), persuading the girl to let her mouth be brushed despite the discomfort. Next, the hygienist directs the patient to open her mouth wider so she can get “it” from the back of the mouth (line 14). While showing discomfort, the girl participates in the activity by remaining still and allowing her teeth to be brushed. However, she closes her mouth on the hygienist’s finger (Figure 4(g)), to which the hygienist responds by gently directing her not to bite (“No, don’t bite, baby” in line 16). The biting movement of the child—most likely an involuntary reaction—directly affects the body of the hygienist by interrupting her work or even hurting her and solicits a defensive verbal response. Thus, mutual touch continues as the patient has agency and the power to touch (even in a way that inflicts pain, i.e., through biting) the hygienist, and therefore the power to resist the institutional action. The bodies, attending to each other’s embodied actions carefully and moment by moment, thus create a close intercorporeal formation of touching and being touched, and affecting and being affected by each other, amid the institutional activity.

Once again, the mother speaks to support the hygienist’s action and to persuade her daughter to let her teeth be brushed (line 17). The hygienist continues portraying her actions as helping to alleviate the patient’s discomfort as she takes a piece of gauze and says, “Here, let me get it out with this towel” (line 18). The patient cooperates, opening her mouth and sticking out her tongue, while the hygienist holds the girl’s arm with her left hand and uses her right hand to wipe the toothpaste off of the girl’s teeth and tongue (Figure 4(h) and (i)). As she wipes the patient’s mouth, the hygienist verbally creates an emotional alliance of “we” against the bad thing in the patient’s mouth when she says in an exaggerated affective tone, “We don’t like that” and “Right? We don’t li::ke it” (lines 19 and 21). Here, again, the hygienist’s talk is relevant to the patient’s resistance to the dental procedure and helps to mitigate the imposition (Cekaite & Bergnehr, 2023) posed thereby by her own sustained, controlling, and discomfort-inducing touch.

Extract 4 demonstrates the crucial role of verbal and vocal action in how touch plays out and affects the participants. Here, the hygienist’s professional touch, including controlling the patient’s body and an instrument-mediated touch causing discomfort or anxiety (brushing the teeth), is framed by the hygienist’s talk: on the one hand, affective words help the hygienist to deal with the patient’s resistance or disengagement, thereby facilitating the continuation of the toothbrushing procedure (Cekaite & Bergnehr, 2023); on the other, words assist the hygienist in conveying a shared emotional stance with the patient against the discomfort- or disgust-causing thing in the mouth. Moreover, in Extract 4, the interaffectivity of touch becomes salient: touching affects both the one who touches and the one who is being touched, and both the hygienist and the patient have power to touch each other.

## Discussion

In this study, we explored the role of touch as a means of helping pediatric dental patients who show signs of distress or fear during dental visits. Drawing on the concept of the intercorporeality (Merleau-Ponty, 1962, 1964, 1968) and the interaffectivity (Fuchs, 2017) of bodies, our detailed video analysis in four different sociocultural contexts (Finland, China, Iraq, and the United States) illustrates that touch in these settings creates a momentary affective (inter)relationship between the bodies touching and being touched: the patient’s embodied affectivity, such as distress, discomfort, or pain, is partially felt by the person who is touching. This fleeting tactile shared experience also enables one body to directly affect another body through various types of tactile actions, such as acts of gentle soothing or of manipulating and controlling.

As a tactile modality, the affective role of touch in health care settings goes “beyond words” (Bjorbækmo & Mengshoel, 2016), especially during medical procedures which tend to cause pain, fear, anxiety, etc. In such settings, verbal resources communicating care, empathy, and reassurance may become “limited, inadequate or unnecessary” (Gleeson & Timmins, 2005, p. 386). Our video analysis of micro-interactions in pediatric dentistry provides rich insights into how various types of touch are mobilized for comforting and controlling a patient in distress. Our examination of Extract 1 showcases comforting touch as a form of empathy (Wyschogrod, 1981); that is, a gentle soothing touch constitutes an interaffective resource for distributing calmness from one body to another. This lends support to Fuchs’ (2017) conceptualization of interaffectivity as providing the basis for primary or intuitive empathic understanding. Our analysis of extended touch in Extract 2 leads to conclusions that align with the findings of Bergnehr and Cekaite (2018) regarding sustained affectionate touch, albeit within a different context. That is, extended comforting touch dissolves the bodily boundary between the self and the other (Guo et al., 2020); using Merleau-Ponty’s (1964) expression, this tactile connection enables the bodies to inhabit a *co-presence*, establishing a state of intercorporeal companionship characterized by a “close and intimate caring social relation” (Bergnehr & Cekaite 2018, p. 953).

Our examination of patient touch (as observed in Extract 3) has yielded results that significantly diverge from numerous prior studies on touch. For instance, previous literature has proposed that health care providers would generally favor initiating touch on patients rather than being initially touched by them (Cocksedge et al., 2013; Edwards, 1998). In Extract 3, however, the patient is granted agency—an ability to touch and not simply be touched—as the assistant asks the child to press her hands into hers firmly while simultaneously holding the child’s hands still. This momentary role reversal, wherein the patient also becomes the initiator of touch, imbues what was primarily a controlling touch with the potential to foster corporeal trust. We have also shown that this reversibility of touching and being touched—the assistant and the patient are simultaneously touching and being touched—constitutes a perfect illustration of “chiasm” proposed by Merleau-Ponty (1968). Finally, in Extract 4, we show the vital role of verbal action in interpreting touch. The hygienist uses empathetic words in conjunction with her controlling and procedural touch, and the verbal action creates a shared emotional stance toward the felt discomfort or disgust. This established emotional affiliation enables the hygienist to persuade the patient to cooperate during the procedure. This finding echoes Cekaite and Kvist Holm’s (2017) conclusion that touch is not merely skin-to-skin or physical (also cf. Estabrooks & Morse, 1992), but importantly, touch unfolds as a multimodal gestalt, involving verbal and vocal action as well as the whole body.

Together, these results paint a nuanced picture of how touch evolves in pediatric dentistry and how it can be used as a resource to help patients during a potentially distressing dental appointment. On the one hand, gentle touch provides an inter-sensorial connection between bodies and, therefore, corporeal reassurance that a body under stress is not alone in the uncomfortable situation. On the other hand, touch also can be more forceful, involving controlling and manipulating tactile actions that can escalate the patient’s negative affect if their ability to control their own body is stripped away from them. In between the gentle and forceful touch, there is a firm touch, often supported vocally, that guides the patient’s bodily movements with the aim of protecting the body from harmful events.

Given that multiple participants often touch the patient at the same time in various ways while also producing different verbal or vocal actions, the interaffectivity of bodies can expand beyond touch and beyond just two bodies. As such, we suggest that rather than treating touch as dyadic, one-way or monomodal action, touch in pediatric dentistry unfolds as complex intercorporeal formations where the interaffectivity emerges not only through touch but also via vocal resonance. Importantly, in contrast to clear boundaries between comforting and controlling touch, our analysis proposes that the line between comforting and controlling touch can be blurred.

To advise the dental practice, we find support for previous studies indicating that gentle touch can help the patients’ to manage through the procedure (Greenbaum et al., 1993). The tactile co-presence of caregivers, such as by holding the patient’s hand or supporting the child’s self-touch, can help to alleviate the fearful situation. Moreover, we witnessed a technique of an assistant letting the patient press her hands amid an uncomfortable situation, and we suspect it gave the child a sense of agency in a situation where her body was controlled by the procedure being done to her. Finally, our analysis suggests that reassuring and empathetic words used during discomfort-causing procedural touch can potentially help to persuade the patient to participate in the operation. Generally, these tactile and multimodal actions fall under established main dental fear and anxiety management techniques, including enhancing trust and control, positive reinforcement, signaling, and relaxation (Armfield & Heaton, 2013). However, the cases we have studied are context-specific, and different patients may react differently to touch. Touching a pediatric patient showing resistance toward a dental procedure requires careful affective attention to the patient’s subtle and moment-by-moment reactions to touch.

## Note

1. Adopting Fuchs’ embodied phenomenology of emotion, affectivity refers to “modes of bodily attunement to, and engagement with, the lived world” (Fuchs, 2013, p. 613). It is only through our affectivity that we find ourselves in a meaningful environment in which persons and things matter for us and in which we care for them as well as for ourselves. “Embodied affectivity consists in the whole interactive cycle which is crucially mediated by the resonance of the feeling body” (Fuchs, 2017, p. 5).

## Appendix 1 The Transcription Conventions Used in the Conversations

**Table:**

(0.5)	Numbers in brackets indicate a time gap in tenths of a second.
(.)	A dot enclosed in brackets indicates a micropause of less than two-tenths of a second.
=	This indicates an absolute contiguity between utterances.
-	This indicates a cut off word.
()	This indicates an unclear utterance or another sound.
:	Colons indicate a stretching of a sound.
.	A full stop indicates a falling tone.
,	A comma indicates a continuing tone.
↑↓	Upward and downward arrows mark the overall rise or fall in pitch across a phrase.
◦ ◦	This indicates a silent voice speech.
?	This indicates a rise in pitch across a word.
Under	This indicates the speaker’s emphasis.
CAPITAL	This indicates the speaker’s extreme emphasis.
@ @	This indicates speech produced with a smiley voice.
(())	This indicates the analyst’s comment.
(H)	This indicates inbreath.
(HX)	This indicates outbreath.
[ ]	This indicates overlapping lines.
|	This indicates the occurrence of embodied actions in relation to talk.
